# International consultation on long-term global health research priorities, research capacity and research uptake in developing countries

**DOI:** 10.1186/s12961-017-0181-0

**Published:** 2017-03-21

**Authors:** David Mc Conalogue, Sue Kinn, Jo-Ann Mulligan, Malcolm McNeil

**Affiliations:** Department for International Development, Abercrombie House, Eaglesham Rd, East Kilbride, Glasgow, G75 8EA United Kingdom

**Keywords:** Global health, Research priorities, Research into policy/practice

## Abstract

**Background:**

In recognition of the need for long-term planning for global health research, and to inform future global health research priorities, the United Kingdom Department for International Development (DfID) carried out a public consultation between May and June 2015. The consultation aimed to elicit views on the (1) the long-term future global health research priorities; (2) areas likely to be less important over time; (3) how to improve research uptake in low-income countries; and (4) how to build research capacity in low-income countries.

**Methods:**

An online consultation was used to survey a wide range of participants on global health research priorities. The qualitative data was analysed using a thematic analysis, with frequency of codes in responses tabulated to approximate relative importance of themes and sub-themes.

**Results:**

The public consultation yielded 421 responses. The survey responses confirmed the growing importance of non-communicable disease as a global health research priority, being placed above infectious diseases. Participants felt that the key area for reducing funding prioritisation was infectious diseases. The involvement of policymakers and other key stakeholders was seen as critical to drive research uptake, as was collaboration and partnership. Several methods to build research capacity in low-income countries were described, including capacity building educational programmes, mentorship programmes and research institution collaboration and partnership.

**Conclusions:**

The outcomes from this consultation survey provide valuable insights into how DfID stakeholders prioritise research. The outcomes from this survey were reviewed alongside other elements of a wider DfID consultation process to help inform long-term research prioritisation of global health research. There are limitations in this approach; the opportunistic nature of the survey’s dissemination means the findings presented may not be representative of the full range of stakeholders or views.

## Background

Since the development of the millennium development goals there have been large improvements in the health of the poorest countries in the world, with reductions in child mortality, new HIV infections, and deaths from malaria and tuberculosis, among others [1]. The epidemiology behind the burden of disease is changing as advances are made in dealing with some of the infectious diseases of poverty [2]. These changes are creating new challenges for donor and recipient countries in how to best invest resources to have the greatest impact on health globally and locally [3, 4]. The Global Goals maintain a focus on important infectious diseases, which still account for the majority of the burden of disease in low-income countries, but also acknowledges the growing importance of other emerging health challenges [4].

Health research is a key tool to deliver change which positively impacts the lives of the poorest, enabling policymakers and practitioners to do more with less resources [5, 6]. To ensure the greatest health impact, it is essential that health research funding priorities and approaches reflect the existing and emerging global health threats to achieve the Global Goals.

While the vast majority of burden of disease globally is based in developing countries, only a small proportion of health research funding is invested in programmes for the benefit of these countries [5]. In recognition of the critical role of research in tackling the determinants of excess mortality and morbidity in low- and middle-income countries, WHO has called on countries to fund research aimed specifically at diseases that affect people in developing countries [7].

The United Kingdom Department for International Development (DfID) supports the importance of investment in research as a key enabler of development, and is the second largest government supporter of product development research [8]. In recognition of the need for long-term planning for funding health research for development and to inform future global health research priorities, DfID carried out a public consultation between May and June 2015. The consultation aimed to elicit views on (1) the long-term future global health research priorities; (2) areas likely to be less important over time; (3) how to improve research uptake in low-income countries; and (4) how to build research capacity in low-income countries.

## Methods

### Participants

Participants were recruited to the consultation survey using established health research networks and social media. Links to the online survey were tweeted from the DfID twitter feed, posted on the DfID website, and current DfID research funding recipients were emailed the survey link. All correspondence encouraged wide sharing of the survey link.

The survey was open for a period of 4 weeks from late May 2015.

### Data collection

Data were collected via Survey Monkey, an online survey tool. Responses to questions were free text, to allow respondents the flexibility to provide a wide range of replies to each question, without being limited to a closed selection of answers. The questions covered the key topic areas of long-term future research priorities, health research areas of reducing priority, research uptake and health research capacity building. For the list of questions, please see Box 1.

Box 1 Consultation survey questions

**Table:** 

What do you think will be the top three priority areas for global health research for the next 20–50 years?
What areas do you think will be less important than today?
In your view, what are the best ways to improve research uptake in low-income countries to get research into policy and practice?
In your view, what are the best ways to build health research capacity in low-income countries?

Respondents were also asked for information regarding their professional affiliation, place of residency (high, middle or low income), and years of relevant work or research experience.

Participation in the survey was voluntary. Individual responses to each question were collated to assure anonymity in data presentation. The only identifiable participant detail taken as part of the survey was their email address (entry was voluntary), which was not linked to survey responses.

### Data analysis

Data were analysed using a thematic analysis, focussing on categories of response provided by consultation responders. The data analysis followed three steps, namely familiarisation with consultation survey responses for individual questions; development of interpretative codes for individual question responses to build a coding framework; and categorisation of codes into themes and subthemes [9].

The analysis also included a tabulation of the frequency of each code from survey responses, to generate an approximation of relative importance of themes and sub-themes within the data.

This consultation process was part of a wider consultation, which also included a health research expert panel, internal DfID health adviser consultation, and Delphi study with expert informants. The outcomes from the consultation exercises were used to inform long-term health research priorities for DfID.

## Results

### Results overview

The public consultation survey yielded 421 responses. The largest proportion of respondents came from academia (43%), which was nearly double the next largest sector, non-governmental organisations (23%). Other respondents came from international organisations (13%), national government (9%) and industry (3%). The majority of respondents came from high-income countries (60%), with 40% from low- or middle-income countries. The relevant experience of respondents ranged from less than 1 year to 50 years, with a modal category of 10 to 15 years of experience.

### What do you think will be the top three priority areas for global health research for the next 20–50 years?

The strongest theme to emerge from the survey responses is the growing importance of non-communicable diseases (NCD). This theme had substantially higher frequency of comment than any other area. The overwhelming focus of responses related to NCD risk factors were on obesity (60 comments), with other risk factors (e.g. diet, hypertension, tobacco and alcohol) receiving lower proportions of responses (no category with a higher frequency than 10 comments). The most frequently mentioned NCDs were cancer (32 comments), diabetes (27 comments), and dementia/neurological conditions (24 comments).

“*Research to inform strategies and policies to prevent and treat non-communicable diseases*”

“*Infectious diseases will continue to be a top priority in global health research, especially the ‘big three’: HIV, TB, and malaria. These diseases remain top killers across the world, and new tools are still urgently needed to curb these epidemics.*” Quotes from participants on long-term future priority areas

While respondents on the whole cited NCDs as the most important research priority, many acknowledge the continuing importance of infectious diseases. The frequency at which NCDs were mentioned compared to infectious diseases possibly reflects the increasing prevalence of NCDs and their associated burden of disease in poor populations, as well as the progress made in infectious disease research during the past decade. Many of the participants referenced infectious diseases as a general category. However, the majority of comments focused on individual diseases, with three subthemes sharing approximately equal frequency; HIV, malaria and emerging diseases.

Other strong themes identified by participants for future research prioritisation include nutrition/malnutrition (114 comments), health systems (95 comments), and maternal, neonatal and child health (79 comments).

### What areas do you think will be less important than today?

There were six times more comments for infectious diseases than any other theme. Within the infectious disease theme, most comments were about specific infectious diseases, with 69 comments for HIV and 32 comments for malaria. There were 39 comments for HIV in the first question (about future priority areas), which possibly reflects recognition of the progress made in HIV and substantial research funding in this area relative to other global health issues. Other themes highlighted were reproductive, maternal, newborn and child health, NCDs, nutrition and vertical programmes, although all received relatively few comments in comparison to infectious diseases.

“*Some of the communicable diseases that have been the greatest priorities over the last 15 years including HIV and malaria may well become less significant in terms of premature death as NCDs … become major causes of death and disability.*” Quote from participant on areas for reducing future importance

### What are the best ways to improve research uptake in low-income countries to get more research into policy and practice?

The involvement of policymakers and other key stakeholders in research processes was highlighted as important to improve research uptake (44 comments). Responses encouraged the early engagement of these groups in the selection of research topics and research approaches. Responses also highlighted the need for continued involvement throughout the research process and into the integration of findings into policy and practice.

The role of collaboration and partnership for evidence uptake was a very strong theme (82 comments), including the importance of collaboration with in-country bodies to integrate evidence into their policy and practice. Research funding as a mechanism to drive evidence uptake was highlighted by participants as a strong driver (63 comments), including research funding criteria on integration of evidence into policy and practice.

“*Embed research into existing* [government] *health programmes so that research addresses national priorities, government has ownership, and research is real-life, leading to real-life answers that are appropriate for the context*.” Quote from participant on how to improve research uptake

Participants also identified key themes for evidence uptake such as education and training aimed at policymakers and researchers, and targeted dissemination of research findings to decision-makers.

### What is the best way to build research capacity in low-income countries?

Informants emphasised the importance of programmes to build researcher capacity (132 comments), including degree or master’s programmes, PhD or post doc programmes, or research scholarships. Within this theme, there was also support for mentorship programmes aimed at early career researchers, with particular attention given to North–South mentorship programmes. Collaboration and partnership was also a strong theme (115 comments), focusing principally on North–South research institution collaboration, although there was also notable support for South–South collaborations to build regional capacity.

“*Build local capacity for research in low-income countries through North–South research collaborations and exchange of research competencies and best practices.*” Quote from participant on how to improve research uptake

The importance of stipulating the inclusion of capacity building as part of the funding decision making was highlighted. Participants also noted the need for researchers to integrate research capacity building into their research design, so that they ensure a lasting legacy beyond programme completion. Capacity building can extend beyond researcher capacity to include in-country policymakers and programme managers. Core to success under this approach is developing research funding opportunities which respond to in-country need, and are more likely to be engaged with by in-country stakeholders.

Investment in the research infrastructure (64 comments), particularly in low-income country research institutions, was also seen as a key method to develop capacity.

## Discussion

DfID used this public survey as part of a wider consultation exercise, which included internal and external sources of feedback, and comprising a Delphi survey of infectious disease experts [10]. The wider process enabled targeted feedback to be combined with the public-facing open access nature of the public consultation. The outcomes of this process were subject to external peer review to validate DfID’s future health research priorities [11]. The approach, of multisource feedback to aid prioritisation of research funding, has the advantage of including a broad set of views, which can be used to inform and influence internal decision-making. This approach was chosen above other systematic approaches, such as the Child Health and Nutrition Research Initiative method [12], since it was felt that such approaches as well as Delphi methodologies tend to focus on expert views, which, while important, negate the inclusion of a wider public stakeholder base, who also have legitimate and important insights into research priorities.

The consultation results provide a valuable insight to both donors and researchers to help direct the long-term development of their respective research portfolios. It also adds to the knowledge base regarding approaches to integrate evidence into policy and practice, and to build research capacity in low-resource settings.

The consultation identified NCDs as the most important research priority for the long-term future. This correlates with the developing burden of disease globally, which shows increasing morbidity and mortality from NCDs in low- and middle-income countries [2, 13], often affecting the poorest people living in low-resource settings [14, 15]. However, there are still many gaps in the evidence base for NCDs in low-resource settings, particularly regarding risk-factors for disease, and which populations are most susceptible [15]. It should also be acknowledged that there is a shifting discourse in global health which recognises the growing importance of NCDs. This is reflected in their prominence in the Global Goals [4] and the development of the World Health Organization Global Action Plan for the Prevention and Control of NCDs [16].

Infectious diseases were identified as both an important theme for future research but also an area to be reduced. This may reflect the recognition of progress made in reducing the burden of infectious disease, and an increasing need to focus on other areas [17]. It could also suggest support for a change in approach to infectious disease research from population wide methods, to concentrating on accessing hard to reach groups and emerging threats. This suggests an important realisation from participants that the important agenda of infectious disease has not been dealt with [10, 18, 19], although there are new emerging threats that should not be overlooked as funders prioritise future investments. Owing to the already very high priority and associated funding given to infectious diseases, it is possibly not surprising that respondents felt this was going to be less important in the future. Nevertheless, this does not preclude support for further investment to consolidate and advance research progress made in this field. The outcomes from the survey regarding increasing and reducing priorities highlight the growing issue of a double burden of disease for infectious diseases and NCDs, which has a stronger impact on the poorest in low-resource settings [20–22].

For research to have a positive effect in low-resource settings, research funding must be based on target country need [23–26]. However, research priorities globally are often based on the requirements of international donor organisations, rather than the recipient countries [25–27]. This potentially leads to research which does not meet the needs of target populations, and where the knowledge generated is not incorporated into policy or practice [28–30]. The results from the consultation support this position, calling for research funding to be based on country need, and to be designed and delivered by the stakeholders in the country of interest.

Our consultation findings also emphasised the importance of early and meaningful engagement of stakeholders in the research process to facilitate effective research uptake. This has to go further than signing up a policymaker to support a research proposal; the key stakeholders must be given influence over the topic selection, research process and outcome implementation. International funding based on the requirements of international donors, rather than the priorities of countries, reduces the impact of research outcomes at a country level [29].

Research capacity building in low- and middle-income countries is essential if solutions to key health issues in low-resource countries are to be progressed [6], although this is a challenging task. Capacity building support for individuals (from a range of backgrounds) was recognised as being key to developing this area in low-resource settings in the consultation responses. Collaboration and partnership was noted as being an important vehicle for this, with particular attention given to North–South institution partnerships. Donors have a responsibility to ensure that research funding places research capacity building as central in funding decision criteria. This requires a more holistic approach, which works at the individual, institutional and the wider research environment [6].

There are limitations in the process and outcomes of the consultation survey. The opportunistic nature of the survey’s dissemination means the findings presented here may not be representative of the full range of stakeholders or views. Additionally, since the consultation was initially distributed by DfID, this may have resulted in a higher response rate from organisations and individuals already affiliated with DfID and may in turn have resulted in responses more in line with current DfID funding priorities and thinking. This was addressed in the wider consultation process, which also included opportunities for targeted feedback. This helped to ensure that opinion from outside of the established DfID relationships could be considered. It should also be noted that the variety of responses in the public consultation, and comparison against current DfID research funding allocations, suggests that respondents represented a far wider spectrum of stakeholders than those who were initially targeted for dissemination of the survey. The terminology used in the consultation questions enquiring to future research priorities uses the terminology ‘global health research’; the authors were keen to ensure as wide a breath of response as possible, but also acknowledge that this terminology may have resulted in quite generic responses. Finally, the thematic analysis will inevitably reflect the values and judgements of the investigators, and the interpretations they have made during and following the process of data analysis. However, by using a systematic and established approach for data analysis, we hope to have reduced inherent bias. Publication of the results also gives other stakeholders the opportunity to present reflections on our interpretation, and to present their own interpretation.

## Conclusions

The above limitations notwithstanding, our view is that these findings, taken together with other sources of qualitative and quantitative information, including those informing the wider consultation, provides valuable insights into how DfID stakeholders prioritise research. The outcomes from this consultation survey have been reviewed alongside the outcomes from the other elements of the consultation to inform the research funding priorities of DfID and wider United Kingdom development funding for global health research [11]. It also provides a useful insight to funders, researchers and research implementers into emerging trends with a global health research focus.
